# Comparative analysis of processed ribosomal protein pseudogenes in four mammalian genomes

**DOI:** 10.1186/gb-2009-10-1-r2

**Published:** 2009-01-05

**Authors:** Suganthi Balasubramanian, Deyou Zheng, Yuen-Jong Liu, Gang Fang, Adam Frankish, Nicholas Carriero, Rebecca Robilotto, Philip Cayting, Mark Gerstein

**Affiliations:** 1 Department of Molecular Biophysics and Biochemistry, Yale University, 266 Whitney Avenue, New Haven, CT 06520, USA; 2 The Saul R Korey Department of Neurology, Albert Einstein College of Medicine, NY 10461, USA; 3 Wellcome Trust Sanger Institute, Wellcome Trust Genome Campus, Hinxton, Cambridgeshire, CB10 1HH, UK; 4 Department of Computer Science, Yale University, New Haven, CT 06520, USA; 5 Program in Computational Biology and Bioinformatics, Yale University, New Haven, CT 06520, USA

## Abstract

**Background:**

The availability of genome sequences of numerous organisms allows comparative study of pseudogenes in syntenic regions. Conservation of pseudogenes suggests that they might have a functional role in some instances.

**Results:**

We report the first large-scale comparative analysis of ribosomal protein pseudogenes in four mammalian genomes (human, chimpanzee, mouse and rat). To this end, we have assigned these pseudogenes in the four organisms using an automated pipeline and make the results available online. Each organism has a large number of ribosomal protein pseudogenes (approximately 1,400 to 2,800). The majority of them are processed (generated by retrotransposition). However, we do not see a correlation between the number of pseudogenes associated with a ribosomal protein gene and its mRNA abundance. Analysis of pseudogenes in syntenic regions between species shows that most are conserved between human and chimpanzee, but very few are conserved between primates and rodents. Interestingly, syntenic pseudogenes have a lower rate of nucleotide substitution than their surrounding intergenic DNA. Moreover, evidence from expressed sequence tags indicates that two pseudogenes conserved between human and mouse are transcribed. Detailed analysis shows that one of them, the pseudogene of RPS27, is likely to be a protein-coding gene. This is significant as previous reports indicated there are exactly 80 ribosomal protein genes encoded by the human genome.

**Conclusions:**

Our analysis indicates that processed ribosomal protein pseudogenes abound in mammalian genomes, but few of these are conserved between primates and rodents. This highlights the large amount of recent retrotranspositional activity in mammals and a relatively larger amount of it in the rodent lineage.

## Abstract 2

An analysis of ribosomal protein pseudogenes in the four mammalian genomes reveals no correlation between number of pseudogenes and mRNA abundance.

## Background

Pseudogenes are DNA sequences similar to genes encoding functional proteins, but are presumed to be nonfunctional due to mutations and truncation by premature stop codons. In this study, we focus on the largest family of pseudogenes, processed pseudogenes of ribosomal proteins (RPs). Previous *in silico *studies have shown that the human genome consists of thousands of processed RP pseudogenes, although there is only one functional gene for each of the 80 human RPs, with the exception of three functional RP retrotransposons [1-5]. The availability of numerous whole genome sequences presents us an opportunity to do a comparative analysis of these pseudogenes in various organisms.

Processed pseudogenes are formed by reverse transcription and integration of processed mRNA into the genome. In the case of human processed pseudogenes, their integration into the genome has been shown to be mediated by L1 transposons and this is believed to be the primary mechanism by which they are generated [6]. We chose to focus on RP pseudogenes because they constitute the largest family of pseudogenes (approximately 2000 RP processed pseudogenes). RP genes are constitutively expressed at reasonably stable levels and are very highly conserved. In addition, RPs have high levels of sequence conservation among various species, which enables us to trace lineages of their pseudogenes easily [7]. The large dataset of RP pseudogenes in conjunction with several completely sequenced genomes allows us to identify orthologous ribosomal pseudogenes in syntenic regions.

Sakai *et al*. [8] estimate that processed pseudogenes are formed at a rate of about 1-2% per gene per million years based on the analysis of processed pseudogenes in human and mouse genomes. Gene duplications occur at a predicted rate of 0.9% per gene per million years in the human genome and are believed to be an important resource for genome evolution. Therefore, they suggest that processed pseudogenes might also play a role in increasing genome diversity, similar to duplication events.

To date, there has been no systematic evaluation of processed pseudogenes in syntenic regions on a large scale. While a study on kinases indicated that processed pseudogenes are not conserved between human and mouse, this study pertains to a very small sample size of about 100 kinase pseudogenes [9]. Suyama *et al*. [10] identified and annotated genes and duplicated pseudogenes under the assumption that processed pseudogenes will not be found in syntenic regions. However, there is no *a priori *reason to expect this. In fact, many studies have identified transcribed processed pseudogenes both by *in silico *methods as well as targeted experimental analyses. Harrison *et al*. [11] analyzed expressed sequence tag (EST) and microarray expression data and came up with a list of about 200 processed pseudogenes that are transcribed in the human genome. The ENCODE consortium experimentally validated transcription of some pseudogenes. They annotated 201 pseudogenes in the ENCODE regions; two-thirds of these pseudogenes were processed. It was shown that at least a fifth of the 201 pseudogenes were transcribed based on pseudogene-specific RACE (rapid amplification of cDNA ends) analyses combined with results obtained from tiling microarray data and high throughput sequencing [12]. Recently, two studies have shown that processed pseudogenes regulate gene expression by means of the RNA interference pathway in mouse oocytes [13,14]. Another study has shown that some ABC transporter pseudogenes are transcriptionally active. They have also shown that the gene expression of an ABC transporter protein is regulated by the expression of its pseudogene in the human genome [15]. Thus, processed pseudogenes are emerging as interesting elements in the genomic landscape capable of being potentially functional.

An elegant study showed that a small number of pseudogenes with high sequence identity to the parent protein are conserved between human and mouse [16]. They suggest that the conservation of sequence in such pseudogenes with high identity to their parent despite being 70 million years old (time of human-mouse divergence) implies a functional role for such pseudogenes. Based on expression evidence and the fact that these conserved sequences are found in syntenic regions between human and mouse, they catalogued a set of 20 pseudogenes that could be potentially functional. The 20 pseudogenes included only two processed pseudogenes that are conserved between human and mouse. The large family of RP processed pseudogenes and the availability of whole genome sequences of many organisms allow us to perform a comprehensive and systematic comparative analysis of RP processed pseudogenes in sytenic regions. It is conceivable that some of them would be conserved across species if they were biologically relevant. RP pseudogenes present a specific problem in that they are often annotated mistakenly as genes due to very high sequence similarity to the parent protein. Here, we use the method developed to identify RP pseudogenes [1], which is elaborated in the Materials and methods section.

For this study, we identified processed RP pseudogenes in four genomes - human, chimpanzee, mouse and rat - using an automated pipeline [17]. We investigated the degree to which processed RP pseudogenes are conserved among the four species. While a significant number of papers have addressed the global synteny between human, chimpanzee, mouse and rat based on DNA sequence alignments, we do not have comprehensive data on detailed local synteny [18-21]. In order to identify well-defined syntenic regions, we defined syntenic regions as sequences conserved in position between orthologous gene pairs. This is similar to the methods used by others where synteny has been derived based on local gene orthology [10,22].

## Results and discussion

### Catalogue of ribosomal protein pseudogenes

In Table 1, we show the total number of RP pseudogenes that occur in each organism. The RP pseudogenes were identified using an established procedure [17] as outlined in the Materials and methods section. All homologous matches with a BLAST e-value more significant than 10^-4 ^were included as potential pseudogenic matches. The pseudogenes have been classified into three groups: processed, fragments, and low confidence matches. Processed pseudogenes are at least 70% long compared to their parent proteins, whereas pseudogenes categorized as fragments have lengths less than 70% of the parent protein. Pseudogenes classified as processed or fragments have a region of homology that has at least 40% amino acid sequence identity to the parent protein with a BLAST e-value <10^-10^. Pseudogenic candidates with a BLAST e -value less significant than 10^-10 ^or with amino acid sequence identity less than 40% of the parent protein are classified as low-confidence matches. Less than 20% of pseudogenes constitute pseudogenic fragments or low confidence matches. This is in accordance with previous studies on all human pseudogenes and RP pseudogenes that showed that the majority of pseudogenes are long [1,23]. We have optimized several parameters in the pseudogene identification pipeline and have obtained a comprehensive catalogue of all pseudogenes. We have included a discussion of the sensitivity of our method for pseudogene identification to changes in parameters as supplementary information in Additional data file 1.

**Table 1:** Total number of processed RP pseudogenes in human, chimpanzee, mouse and rat genomes identified by the pipeline [17]

Organism	Processed	Fragment	LC
Human	1,822	218	212
Chimpanzee	1,462	219	160
Mouse	2,092	326	413
Rat	2,848	343	450

LC, low confidence matches.

The number of processed pseudogenes associated with each RP for the four organisms is shown in Additional data file 2. Our analysis is primarily focused on the major group of pseudogenes, processed pseudogenes that are at least 70% long compared to their parent proteins. Calculations that included pseudogenic fragments and low confidence matches did not affect the comparative results obtained [1,23]. Moreover, we are interested in identifying candidate pseudogenes that are exceptionally well conserved over a long time period. It is clear that all four genomes are replete with processed RP pseudogenes. The human, chimpanzee, mouse and rat genomes contain 1,822, 1,462, 2,092 and 2,848 processed RP pseudogenes, respectively. The length of coding sequence associated with each human RP gene is included in parentheses in Additional data file 2; these clearly show that the number of pseudogenes arising from a RP gene is not influenced by mRNA length. Our assignments can be downloaded from [24]. The number of pseudogenes per RP varies dramatically from a few in number to over a hundred in some cases. The higher number of processed RP pseudogenes in rat and mouse may reflect the reported higher rates of retrotranspositional activity in the rodent lineage [18,20].

### Analysis of expression levels

**Figure 1:**
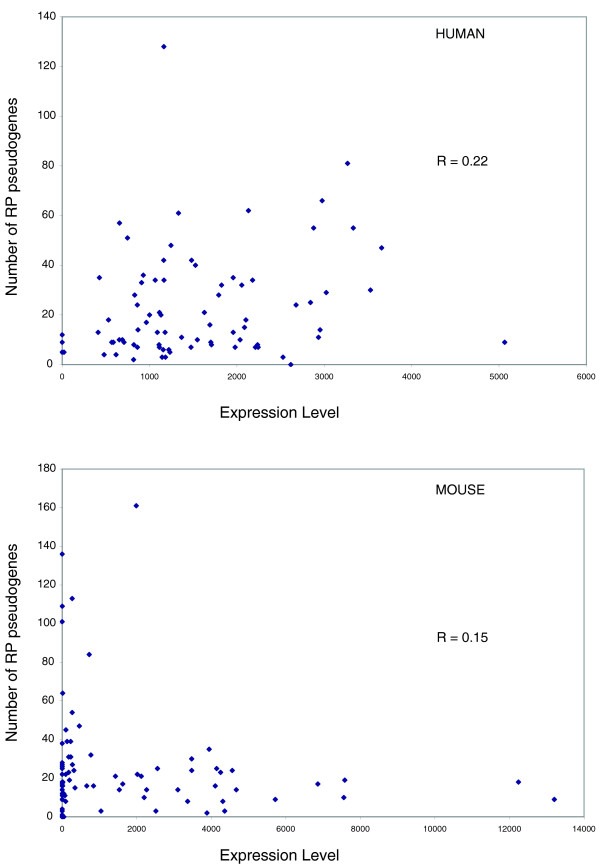
Plot of expression level of mRNA in testes associated with each RP protein versus the number of processed pseudogenes associated with it. The top and bottom panels correspond to human and mouse RP pseudogenes, respectively. The x-axis shows signal on the gene chip, which is a measure of the abundance of a mRNA transcript. Data for the human and mouse are not normalized to each other and should not be compared directly. It should be noted that expression data for some RP proteins for mouse are missing in the GEO data.

**Figure 2:**
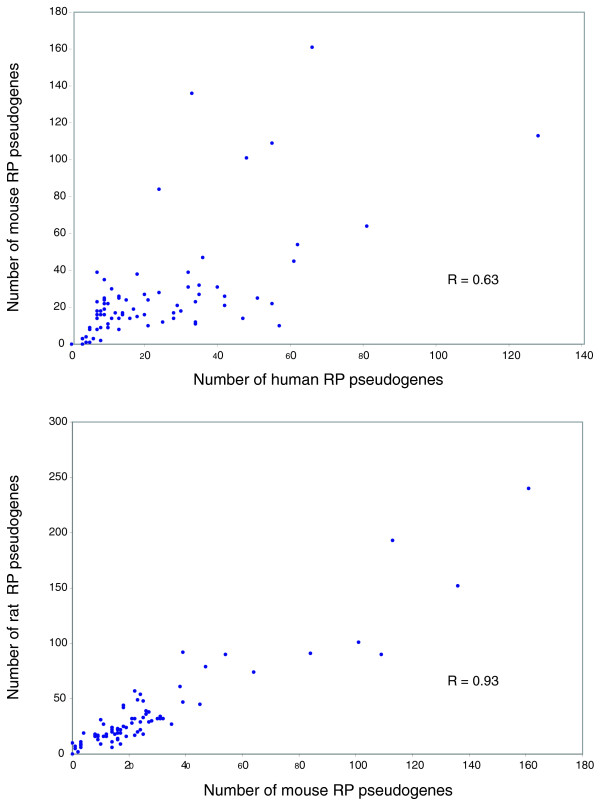
Plots depicting the number of processed pseudogenes associated with a RP protein in one organism and its corresponding ortholog in another organism. The top panel shows the comparison between human versus mouse and the bottom panel depicts the same for mouse versus rat RP pseudogenes. Each point corresponds to the number of processed RP pseudogenes associated with one RP in the two species that are being compared.

Previously, it has been shown that house-keeping genes generally have more processed pseudogenes [25]. Higher mRNA levels of housekeeping genes relative to other genes could help explain the greater number of their corresponding processed pseudogenes. Therefore, we correlated mRNA expression levels of the RPs to the number of pseudogenes per protein. Surprisingly, we did not observe any obvious correlation between the mRNA level for a RP gene and the number of pseudogenes derived from it in both the human and mouse samples (Figure 1; R = 0.22 and 0.15 for the human and mouse expression data sets, respectively). Similar results were reported earlier using yeast and unpublished human expression data sets [1]. Our analysis is based on a more recent expression data set that includes RP mRNA abundance from human and mouse testes [26]. This suggests that expression level is not the only dominant factor determining the number of pseudogenes arising from a gene. However, we have to be cautious about interpreting these results. The discrepancy between mRNA expression levels and the number of pseudogenes associated with a RP could be attributed to unreliability in measurement of mRNA levels due to contamination from somatic cells as well as due to varying mRNA stabilities as proposed by Pavlicek *et al*. [27]. On the other hand, when we examined the numbers of processed pseudogenes per RP across multiple species, we see that the same parent protein seems to have similar numbers of processed pseudogenes in each organism. Figure 2 shows a plot of the number of processed pseudogenes associated with each RP in human versus mouse and the corresponding data for mouse versus rat. The number of processed pseudogenes per RP is very well correlated for the rat versus mouse comparison (R = 0.93). A similar comparison of human versus mouse RP pseudogenes shows a smaller but significant correlation (R = 0.63). This indicates that there may be a relationship between the underlying sequence composition of the parent RP gene and retrotransposition regardless of the expression level of each gene, leading to similar retrotranspositional activity in the primate versus rodent lineage.

### Identification and analysis of syntenic pseudogenes

**Figure 3:**
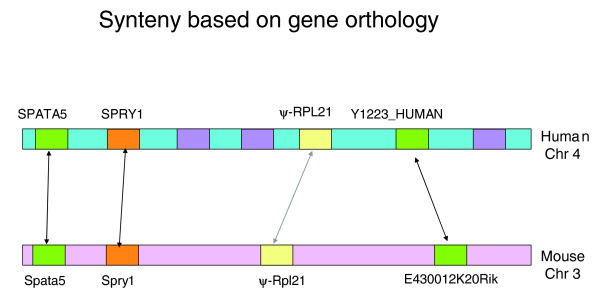
Schematic representation of the method used to identify syntenic regions between two species. In this figure, the pseudogenes are depicted as yellow boxes and human genes that have orthologs in mouse have been labeled. As explained in the text, the human gene *SPRY1 *and Y1223_HUMAN sandwich the processed RP pseudogene of *RPL21 *and have corresponding orthologs in the mouse genome. Thus, we identify this region as being syntenic between human and mouse. Orthologs were identified based on annotations from Ensembl release 36.

We identified RP pseudogenes that are in syntenic regions using the methodology outlined in the Materials and methods section and in Figure 3. Essentially, we identified orthologous genes between two species and identified the regions sandwiched between pairs of orthologous genes as syntenic regions.

Table 2 contains the results of the synteny analysis. From Table 2, it is clear that a significant portion of processed RP pseudogenes is preserved between the human and chimpanzee genomes whereas there is almost no preservation of RP pseudogenes between human and the rodent lineage. The recent divergence between human and chimpanzee explains the high level of preservation of pseudogenes between the two species and that the shared RP pseudogenes were generated before the split of human and chimpanzee. Of the 1,462 RP pseudogenes identified in the chimpanzee genome, 1,282 are preserved between human and chimpanzee. Thus, 87% of RP pseudogenes are conserved between humans and chimpanzees. While it is true that the human and chimpanzee genomes are very similar, the slightly lower number of conserved RP pseudogenes than expected can be attributed to a variety of factors, including a 3% indel difference between the two species and the poorer quality of the chimpanzee genome sequence. The low level of conservation between human and rodents indicates that either the ancestral pseudogenes have decayed significantly or most of the pseudogenes in human and rodents are lineage-specific [9,10]. All the data pertaining to these syntenic pseudogenes can be downloaded from [24].

**Table 2:** Number of processed RP pseudogenes found in syntenic regions

Species1-species2	Number of processed RP pseudogenes in syntenic regions
Human-chimpanzee	1,282
Human-mouse	6
Human-rat	11
Mouse-rat	394

### Sequence divergence of pseudogenes

**Figure 4:**
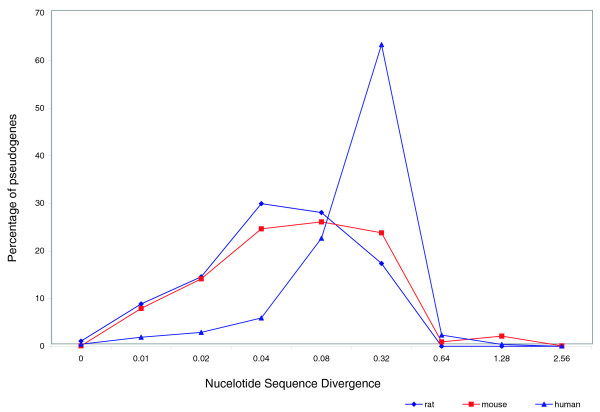
Processed pseudogenes grouped according to their nucleotide sequence divergence from the parent RP protein. The distances have been calculated using MEGA [28]. The distance is a measure of the number of nucleotide substitutions per site. For mouse and rat, the distances have been scaled by decreasing it by a factor of three based on the reported observation that a threefold-higher rate of base substitution in neutral DNA is found along the rodent lineage when compared with the human lineage [20].

We calculated the sequence divergence between a pseudogene and its parent gene using MEGA [28]. Figure 4 shows the distribution of RP pseudogenes as a function of nucleotide sequence divergence between a pseudogene and the parent gene for the human, mouse and rat genomes. It is known that rodents have a higher neutral substitution rate compared to other mammals. It has been speculated that this is due to their shorter generation time [29]. With the availability of the human, mouse and rat genomes, the rat genome consortium calculated the neutral substitution rates based on a comparison of ancient repeats in these three genomes [20]. They showed that the base substitution in neutral DNA is approximately threefold higher in rodents than in humans and, therefore, the divergence distances for mouse and rat have been scaled accordingly [20]. From Figure 4, it is clear that the overall distribution is different for the human versus rodent lineage. The mouse and the rat curves look very similar to each other. RP pseudogenes in mouse and rat are predominantly of recent origin (lesser divergence distance). The absence of any significant preservation of processed RP pseudogenes between human and mouse indicates that most processed RP pseudogenes in both human and rodent lineages are of recent origin, presumably formed after the human-rodent split.

### Nucleotide substitution analysis

#### Human-mouse comparison

We calculated the number of nucleotide substitutions in the syntenic pseudogenes between human and mouse by aligning pairs of conserved syntenic pseudogenes. We also performed a similar calculation for the intergenic DNA surrounding the pseudogenes. The results are indicated in Table 3. It is clear that the syntenic pseuodgenes have a much lower number of substitutions per site than their surrounding DNA. Moreover, EST data indicate that one of these, a pseudogene of *RPS27*, is transcribed in both human and mouse, and for another, a pseudogene of *RPL29*, there is transcriptional evidence for the human *RPL29 *pseudogene. The lower substitution rate seen in syntenic pseudogenes coupled with some transcriptional evidence is suggestive of a possible biological role for the conserved syntenic pseudogenes between human and mouse.

**Table 3:** Comparison of number of nucleotide substitutions per site between pseudogenes and intergenic sequences in syntenic regions of human and mouse

RP protein	Human chromosomal location	Mouse chromosomal location	Pseudogenes	Intergenic regions	EST evidence
RPL21	4:125024510:125024986:-	3:37423214:37423683:-	0.292	1.082	--
RPL29	8:49459705:49460174:+	16:13988323:13988790:-	0.374	1.205	+-
RPL35A	4:164660936:164661273:+	8:65697845:65698079:-	0.312	1.101	--
RPL7A	18:35168834:35169634:-	18:26052080:26052856:-	0.123	1.098	--
RPS27	15:61234862:61234984:-	9:67074892:67075023:+	0.159	1.137	++

The chromosomal coordinates are indicated as follows: 'Chromosome number:Start:End:Strand'. For the EST evidence column, the first symbol denotes transcription in human and the second symbol transcription in mouse; a plus sign (+) indicates evidence of transcription and a minus sign (-) indicates absence of transcriptional evidence.

Careful manual analysis of the human-mouse syntenic pseudogenes indicates that the pseudogene of *RPS27 *is very likely to be a functional protein-coding gene (*RPS27L*) highly similar to *RPS27*. The proteins encoded by human *RPS27 *and *RPS27L *are the same length (84 amino acids) and differ at only three residues (5, 12 and 17). The similarity of these two loci at the amino acid level suggests that either *RPS27 *or *RPS27L *arose via duplication of the other locus. This is further supported by the arrangement of flanking genes; both *RPS27 *and *RPS27L *are flanked on one side by RAS oncogene family genes (*RAB13 *for *RPS27*, *RAB8B *for *RPS27L*) in the same tail to tail arrangement. However, genes on the other flank are different (nucleoporin 210 kDa-like (*NUP210L*) for *RPS27*, lactamase, beta (*LACTB*) for *RPS27L*) and intronic conservation is very low. Very low conservation of intronic and flanking sequence suggests that any duplication event was not recent and this is supported by the conservation of synteny; *LACTB*/*RPS27L*/*RAB8B *is conserved in chimp, macaque, mouse, dog, cow and monodelphis (but not rat, chicken, *Xenopus *or zebrafish) and *RAB13*/*RPS27*/*NUP210L *shows a very similar pattern of conservation (although this synteny is conserved in rat). Further support for function comes from the strong evidence of transcription at the *RPS27L *locus, which is seen in both the human and mouse genomes as well as other vertebrates (Figure 7 in Additional data file 1). This is a significant finding because eighty ribosomal proteins in the human genome have been carefully mapped and the *RPS27*-like gene has not been identified in this study [3]. The comprehensive Ribosomal Protein Gene database, which catalogues RP data for several organisms, does not include this gene [7]. Thus, this serendipitous finding provides the basis for further experimental study of the *RPS27L *locus.

#### Human-chimpanzee comparison

Of the 1,282 human-chimp pseudogne pairs found in syntenic regions, 545 pairs are found within introns of genes. After excluding this group of intronic pseudogenes, we calculated the number of nucleotide substitutions per site in pseudogenes and the intergenic DNA surrounding the pseudogenes. The average number of substitutions per site since the human-chimpanzee divergence is 0.020 and 0.075 in pseudogenes and intergenic regions, respectively. Substitutions in pseudogenes are significantly slower than their neighboring intergenic sequences (*p *<< 0.001, pairwise *t*-test). We find that the pseudogenes evolve slower than the surrounding intergenic DNA. This implies that the pseudogenes conserved in human and chimpanzee might be under some biological constraint.

### Analysis of decayed pseudogenes

It has been noted that 22% of the human genome is composed of ancient repeats, in contrast to a corresponding number of 5% in the mouse genome [18]. It has been rationalized that the fast mutation rates in mouse makes such sequences undetectable. Therefore, it is difficult to identify very decayed pseudogenes. Previous studies indicate that our method used to identify pseudogenes in the human genome is fairly robust and that the cutoffs chosen for various parameters are optimal [23]. We have performed a similar analysis for the mouse genome. Our results indicate that we have comprehensively identified all the pseudogenes in the mouse genome (data included in Additional data file 1). In our current analyses, less than 20% of RP pseudogenes are classified as either fragments or low confidence matches in human, chimp, mouse and rat genomes (Table 1). Thus, only a very few ribosomal pseudogenes represent substantially decayed pseudogenes. Nonetheless, we analyzed human and mouse pseudogenic fragments to ensure the inclusion of older pseudogenes that would have decayed significantly in our analysis. Of the 326 mouse pseudogenic fragments, only one has a corresponding human pseudogene in syntenic regions. None of the low confidence matches in human and mouse genomes had corresponding pseudogenic matches in syntenic regions. Thus, the analyses of all classes of pseudogenes - the longer processed pseudogenes (length ≥ 70% of parent protein), pseudogenic fragments (length <70% of parent protein) and the low confidence matches - indicate that there is very little preservation of processed RP pseudogenes between human and mouse.

## Conclusion

We have systematically analyzed the conservation of processed pseudogenes across four species by looking at a large family of RP processed pseudogenes in syntenic regions. This is the first large-scale comparative analysis of processed pseudogenes. This analysis indicates that while processed RP pseudogenes abound in both human and rodent species, there is virtually no preservation of processed RP pseudogenes between human and rodents. The divergence of RP pseudogenes from their parent genes indicates that most pseudogenes in rodents are of recent origin. This is in line with the reported increased retrotranspositional activity in rodents relative to humans and in accordance with research that indicates that retrotransposition in the hominid lineage has decreased significantly over the past 40 million years [18,30-32]. Our result is also consistent with the previous report that showed that about 80% of all human processed pseudogenes are primate-specific sequences [12]. We did not detect older RP pseudogenes that may have originated from a common ancestor to man and mouse due to faster neutral substitution and higher deletion rates in rodents. Our analyses show that either RP processed pseudogenes present in the human-rodent ancestors have been deleted in current human and mouse/rat genomes or they have decayed significantly beyond recognition by our methods. The RP pseudogenes detected by our methods are predominantly of recent origin and arose by independent lineage-specific retrotranspositional activities. Interestingly, both in the case of human-mouse and human-chimpanzee, the syntenic processed RP pseudogenes appear to have evolved slower than neutral DNA. This is suggestive of a potential biological role for the conserved syntenic pseudogenes. EST evidence of transcription in both human and mouse, together with strong conservation of exons and evidence of transcription in many vertebrates, indicates that *RPS27L*, identified as a pseudogene, is likely to be a functional gene.

## Materials and methods

### Synteny based on gene orthology

We derived syntenic regions based on the criterion that syntenic regions in two species should have corresponding orthologs of genes on the two sets of chromosomes. We obtained syntenic blocks based on gene orthology between two organisms as follows: first, we located the genes on either side of a pseudogene; second, we identified the corresponding orthologous genes in the second organism - the human gene annotations and their ortholog annotations in the other organisms were directly extracted from Ensembl release 36 [33]; third, the region encapsulated between the two sets of orthologous genes on either side of the pseudogene constitutes a syntenic block.

Figure 3 illustrates the methodology used to define syntenic regions between human and mouse. This method defines syntenic regions rather conservatively. To make it less restrictive, we did not constrain the search to include only immediate neighboring genes. We allowed any two regions to be syntenic provided the RP pseudogene was sandwiched between a set of orthologous gene pairs on either side. This means that as long as we were able to find a pair of orthologous genes on either side of the pseudogene irrespective of any number of intervening genes with no orthologs in the other organism, we still defined it as a syntenic block. Thus, this method does not take into consideration potential loss of local synteny due to recombination and chromosomal rearrangements. Recombination rates are non-uniform across the genome and vary depending on the species [34]. Moreover, segmental duplications of varying nature in different species will also affect synteny mapping [35]. Despite these limitations, control calculations designed to test how well random genomic DNA could be located between orthologous gene regions showed that large scale synteny is largely preserved, similar to the earlier large scale genome-wide alignments [18]. We validated this method using two different controls as discussed below.

First, we evaluated how well this method performed by identifying orthologous RP genes between human and mouse in syntenic regions. Of the 79 orthologous RP genes, 76 were identified in syntenic regions. Thus, 96% of the RP genes were identified in syntenic regions. Second, we also looked at the occurrence of 1,000 bp DNA sequences extracted randomly from the genome in syntenic regions to evaluate the extent to which chromosomal rearrangements might affect the identification of syntenic blocks. We chose 1,000 bp regions from the chimp and mouse genomes and identified syntenic blocks around these regions. We found 94% and 86% of such randomly chosen 1,000 bp regions from the chimp and mouse genomes, respectively, to be syntenic to the human genome. A similar control calculation also showed that 86% of randomly chosen 1,000 bp mouse regions were found in syntenic regions of the rat genome. Sample sizes >10,000 were used for these validations. These results indicate that a significant portion of the genomes can be found in syntenic blocks and the errors that might arise due to chromosomal rearrangements are small. Thus, this method of finding syntenic blocks based on gene orthology is fairly robust and provides a good way to identify pseudogenes in syntenic regions.

### Identification of processed RP pseudogenes

We identified processed RP pseudogenes in four organisms - human, chimpanzee, mouse and rat - using a well-established automated pipeline for identification of pseudogenes [1,17]. In a nutshell, this involves identification of pseudogenes based on sequence homology to RPs. The pipeline procedure was modified a little as described here. One of the pipeline steps uses gene annotations to filter out genes from pseudogene candidate sequences. Many RP pseudogenes are often mistakenly annotated as genes in gene annotation databases, including Ensembl [23], and because there are an unusually large number of processed RP pseudogenes, most of them are highly similar to their parent protein. Therefore, we decided to use pseudopipe without reference to RP gene annotations from Ensembl. Instead, we used RP sequences from the Ribosomal Protein Gene database as input and considered the RP genes annotated in this database as the only functional genes [7]. The human, chimp, mouse and rat genome versions corresponding to the assembly in Ensembl release 36 were used as input for the pipeline.

### Expression analysis

The mRNA abundances of ribosomal proteins in the human and mouse testes were obtained from the Gene Expression Omnibus [GEO:GSE1133] [26,36].

### Evolutionary distance

We calculated the nucleotide sequence divergence between the parent RP gene and each pseudogene using the evolutionary analysis package MEGA3 [28]. We calculated the evolutionary distance between the parent RP gene and each pseudogene following the Kimura 2-parameter model [37]. The distance is a measure of the number of nucleotide substitutions per site.

### Nucleotide substitution analysis for syntenic pseudogenes

We calculated the number of nucleotide substitutions per site since the human-chimpanzee divergence and human-mouse divergence for each pair of corresponding syntenic pseudogenes using the Kimura 2-parameter model [37]. Pairs of syntenic pseudogenes between human and chimpanzee and human and mouse were aligned by ClustalW for this analysis [38]. We also performed similar calculations on intergenic DNA by aligning 10 kb of intergenic DNA surrounding the syntenic pseudogene on either side. Gaps in alignments were regarded as transversions for this analysis, where only the first gap in an indel was included and the rest were not counted. For this analysis, we excluded pseudogenes that are within introns of genes as intronic sequences are known to be conserved [39] and would not serve as a good model for neutrally drifting DNA.

### Evidence for transcription

We used EST data from dbEST for verifying if human and mouse pseudogenes in syntenic regions are transcribed [40]. For evidence of transcription, we required a stringent 100% sequence identity of the EST transcripts to the matched region. In cases of less than 100% sequence identity, we required that the EST match the pseudogene better than the parent gene or any other region in the genome.

## Abbreviations

EST: expressed sequence tag; RP: ribosomal protein.

## Authors' contributions

SB performed the bioinformatic analyses, DZ, YL, GF, RR and PC helped with various details of the analyses, AF performed manual analyses of syntenic pseudogenes in human and mouse, and NC provided pseudogene assignments using PseudoPipe. This work was performed in the laboratory of MG. All authors read and approved the final manuscript.

## Additional data files

The following additional data are available with the online version of this paper. Additional data file 1 includes details on the sensitivity of our method for pseudogene identification and the detailed analysis of one of the human-mouse syntenic pseudogenes that appears to be a protein-coding gene. Additional data file 2 includes a table showing the number of processed pseudogenes associated with each RP gene for human, mouse, chimpanzee and rat.

## Supplementary Material

Additional data file 1 Figures 5 and 6: the variation in the number of pseudogenes identified when the percent identity cutoff and e-value cutoff is varied. Figure 7: the results of manual annotation of the *RPS27L*/*Rps27l *locus in human and mouse. Click here for file

Additional data file 2 Processed pseudogenes associated with each RP gene for human, mouse, chimpanzee and rat. Click here for file
