# Supplementary material for: Comparative analysis of processed ribosomal protein pseudogenes in four mammalian genomes
Source: Genome Biol. 2009 Jan 5;10(1):R2. doi: 10.1186/gb-2009-10-1-r2 (PMC2687790; doi:10.1186/gb-2009-10-1-r2)
Supplement: Additional data file 1 — Figures 5 and 6: the variation in the number of pseudogenes identified when the percent identity cutoff and e-value cutoff is varied. Figure 7: the results of manual annotation of the RPS27L/Rps27l locus in human and mouse. [file gb-2009-10-1-r2-S1.doc]

**Identification of mouse pseudogenes using our pipeline**

We varied the percent identity and e-value cut-off for pseudogene identification using our pipeline. For the mouse genome, the total number of pseudogenes varied between 16,964 – 15,884 when the percent identity cutoff of the pseudogenic match to the region of homology of the parent protein was varied between 25 to 50%. This is shown in Fig 5. Thus, we see that the number of pseudogenes do not change substantially when we vary the sequence identity parameter. Please note that this analysis pertains to all mouse pseudogenes and not just mouse RP pseudogenes. The number of pseudogenes at 40% identity cutoff is 16,730.

We performed a similar analysis by varying the e-value cutoff that we use to assign a potential BLAST pseudogenic match to a parent protein. As the e-value is lowered, the number of significant matches drops as expected. At an e-value cut-off 10^-10^, 16,730 mouse pseudogenes are identified. When the cut-off is raised to e-value of 1, the total number of pseudogenes increases only to 19,730 (Figure 6a). Thus, an e-value cut off 10^-10^ captures high confidence matches. Figure 6b shows that as e-value cut-off is changed between 10^-10^ to 10^-1^, number of pseudogenes identified plateaus at an e-value cut-off of 10^-4^. By analyzing pseudogenes with e-value less than 10^-4^ (the low confidence matches), we have made sure we have included all the pseudogenes that can be identified reliably. Thus, we have comprehensively identified all the pseudogenes using our pipeline and have shown that our choice of parameters includes significantly decayed pseudogenes.

Figure 5

Figure 6a

Figure 6b

Figure 7

Manual annotation of RPS27L/Rps27l locus in human and mouse.

Panel A shows a screenshot of the Zmap annotation interface for the human RPS27L locus (Harrow et al. GENCODE: producing a reference annotation for ENCODE. Genome Biol. 2006;7 Suppl 1:S4.1-9. Epub 2006 Aug 7. PMID: 16925838). Annotated coding gene objects are shown in open red boxes (5' and 3' UTRs) and open green boxes (CDS portion of transcripts); non-coding transcripts are represented as solid red boxes. The open grey boxes show represent a Pfam domain (Ribosomal protein S27, PF01667.1) identified by the halfwise algorithm (http://www.ebi.ac.uk/Tools/Wise2/index.html). The majority of transcription starts within a CpG island (solid yellow box). There is a large amount of evidence for locus-specific transcription provided by human ESTs (solid purple boxes) and vertebrate mRNAs (solid brown boxes). Green connecting lines indicate contiguity between blocks of homology. Red dots indicate unaligned terminal sequence.

Panel B shows a screenshot of the Zmap annotation interface for the mouse Rps27l locus. As in the case of the human counterpart, a Ribosomal protein S27 Pfam domain was identified, most transcription initiates within a CpG island and there is a large amount of evidence for locus-specific transcription provided by mouse ESTs and vertebrate mRNAs.
