# Supplementary material for: Comparative analysis of processed ribosomal protein pseudogenes in four mammalian genomes
Source: Genome Biol. 2009 Jan 5;10(1):R2. doi: 10.1186/gb-2009-10-1-r2 (PMC2687790; doi:10.1186/gb-2009-10-1-r2)
Supplement: Additional data file 2 — Processed pseudogenes associated with each RP gene for human, mouse, chimpanzee and rat. [file gb-2009-10-1-r2-S2.rtf]

Table 4

Number of processed RP pseudogenes for each RP protein in human, chimpanzee, mouse and rat genomes identified by the pipeline [17]

The CDS of the human RP gene is given in parenthesis in column 1. The absence of any pseudogenes for RPL41 is probably due to the short length of the protein making it difficult to identify hits for this RP protein in BLAST runs. The pseudogenes are at least 70% as long as the parent protein.

Ribosomal protein (CDS)	Human	Chimpanzee	Mouse	Rat	
					
RPL21 (483)	128	108	113	193	
RPL23A (471)	81	64	64	74	
RPL7A (801)	66	52	161	240	
RPL31 (378)	62	55	54	90	
RPSA (888)	61	52	45	45	
RPS26 (348)	57	34	10	31	
RPL7 (747)	55	48	22	57	
RPS2 (882)	55	48	109	90	
RPL36A (321)	51	39	25	48	
RPL17 (555)	48	41	101	101	
RPS3A (795)	47	32	14	11	
RPL12 (498)	42	31	21	32	
RPL39 (156) 	42	33	26	39	
RPS15A (393)	40	36	31	32	
RPL35A (333)	36	32	47	79	
RPL26 (438)	35	25	32	32	
RPL32 (408)	35	27	27	29	
RPL34 (354)	34	27	23	49	
RPL5 (894)	34	29	12	18	
RPS20 (360)	34	29	11	27	
RPL29 (474)	33	22	136	152	
RPL9 (579)	32	24	39	92	
RPS12 (399)	32	29	31	34	
RPS10 (498)	30	24	18	44	
RPS27 (255)	29	21	21	28	
RPL6 (867)	28	23	17	19	
RPS29 (171)	28	20	14	24	
RPL13A (612)	25	17	12	16	
RPL37 (294)	24	18	28	30	
RPS6 (750)	24	21	84	91	
RPL19 (591)	21	18	24	54	
RPL22 (387)	21	11	10	9	
RPL15 (615)	20	15	27	38	
RPS4X (792)	20	18	16	14	
RPL36 (318)	18	19	38	61	
RPS24 (402)	18	11	15	18	
RPS27A (471)	17	12	19	24	
RPS17 (408)	16	13	14	22	
RPL30 (348)	15	16	24	29	
RPL18A (531)	14	10	16	13	
RPS18 (459)	14	14	17	22	
RPL10A (654)	13	11	8	18	
RPL13 (636)	13	11	26	36	
RPL18 (567)	13	9	14	20	
RPS7 (585)	13	10	25	33	
RPLP1 (345)	12	11	17	9	
RPL27 (411)	11	10	30	32	
RPL3 (1212)	11	11	14	6	
RPL23 (423)	10	8	11	16	
RPS14 (456)	10	8	9	13	
RPS16 (441)	10	9	9	16	
RPS25 (378)	10	7	22	32	
RPL10 (645)	9	6	24	22	
RPL37A (279)	9	8	25	18	
RPL40 (387)	9	7	22	17	
RPLP0 (954)	9	7	19	16	
RPS15 (438)	9	5	16	19	
RPS8 (627)	9	7	35	27	
RPL35 (372)	8	7	18	25	
RPS13 (456)	8	7	16	23	
RPS28 (210)	8	4	9	17	
RPS5 (615)	8	7	2	2	
RPL24 (474)	7	8	14	24	
RPL27A (447)	7	4	39	47	
RPS11 (477)	7	6	23	20	
RPS19 (438)	7	6	18	42	
RPS21 (252)	7	7	8	16	
RPS23 (432)	7	6	16	22	
RPL4 (1284)	6	6	3	8	
RPS3 (732)	6	6	3	9	
RPL11 (537)	5	5	9	16	
RPL28 (414)	5	4	8	17	
RPLP2 (348)	5	3	1	5	
RPL14 (648)	4	2	1	7	
RPS9 (585)	4	2	4	19	
RPL38 (213)	3	3	3	11	
RPL8 (774)	3	3	3	6	
RPS30 (402)	3	2	0	10	
RPS4Y (792)	2	1	0	0	
RPL41 (78)	0	0	0	0	
Total	1822	1462	2092	2848	
